# CT-derived FFR: beyond anatomy

**DOI:** 10.1007/s12471-023-01763-6

**Published:** 2023-01-26

**Authors:** Coen K. M. Boerhout, Jan J. Piek

**Affiliations:** Department of Cardiology, Heart Centre, Amsterdam University Medical Centres, Amsterdam, The Netherlands

New-onset stable angina is a frequently encountered problem in current clinical practice and the most common manifestation of obstructive coronary artery disease (CAD). From its very first presentation, treating cardiologists have to choose either conservative, optimal medical therapy or invasive treatment with the option of revascularisation. Consequently, the diagnostic management is centred on the selection of patients who may benefit from revascularisation therapy. Coronary computed tomography angiography (CCTA) was introduced as a noninvasive and easily accessible alternative for invasive coronary angiography to determine the presence of obstructive CAD. However, although CCTA has an excellent negative predictive value, its positive predictive value for haemodynamically significant lesions is poor [1]. Consequently, an important limitation of widespread implementation of CCTA is the potential increase of unnecessary invasive coronary angiographies.

The addition of haemodynamic assessment of coronary lesions by means of CCTA-derived fractional flow reserve (FFRct) may overcome this shortcoming. Early validation studies and observational data have shown a promising effect as FFRct improves the diagnostic accuracy of CCTA as a gatekeeper and safely defers patients from invasive interventions. Additionally, the FUSION trial, as presented by Sharma et al. in the current issue of the *Netherlands Heart Journal*, will investigate the role of FFRct in a multicentre, randomised, controlled trial [2]. Since this is a Dutch study, the design was welcomed to be published in our national journal. It is expected that the outcomes of this study will contribute to the discussion on how to manage the noninvasive assessment of patients with new-onset stable angina.

Yet, some aspects still merit consideration and should be included in the interpretation of the expected results. The first goal of revascularisation therapy in patients with stable obstructive CAD is symptom reduction. The identification of ‘unnecessary’ invasive coronary angiography should be optimally determined based on patient-reported outcomes. Secondly, it is important to note that FFR, either invasively or noninvasively derived, does not describe the complete haemodynamic properties of a coronary lesion. Simply stated, the pressure drop across a stenosis is proportionally related to the flow through it. High-flow values across a minor lesion may exhibit abnormal FFR values, and vice versa, low-flow values (i.e. in the presence of microvascular dysfunction) may falsely designate a severe lesion as normal.

In addition, recent studies in patients with stable and unstable coronary syndromes (i.e. FUTURE, LOWER-MI and RIPCORD-2) showed that an FFR-guided revascularisation strategy does not improve clinical outcomes compared with an angiography-guided strategy [3–5]. Therefore, validation studies of new parameters, such as FFRct, by comparison with FFR are no longer sufficient. Rather. these new parameter-based strategies should be evaluated in a randomised fashion against angiography. As the design of the randomised FUSION trial allows comparison of an FFRct arm with a usual-care arm, this study may provide novel insight into the usefulness of FFRct for patient management.

In conclusion, the FUSION trial is an important study as it is expected that use of FFRct may further reduce the number of unnecessary coronary interventions.
